# Serum Levels of Copper, Ceruloplasmin and Angiotensin Converting Enzyme among Silicotic and Non-Silicotic Workers

**DOI:** 10.3889/oamjms.2015.065

**Published:** 2015-06-16

**Authors:** Safia Beshir, Hisham Aziz, Weam Shaheen, Eman Eltahlawy

**Affiliations:** *National Research Centre, Environmental and Occupational Medicine Department, Cairo, Egypt*

**Keywords:** Serum copper, ceruloplasmin, ACE, silicosis

## Abstract

**BACKGROUND:**

Silicosis is the most frequently occurring pneumoconiosis.

**AIM:**

Measurement of serum levels of Angiotensin converting enzyme (ACE), Copper (Cu) and Ceruloplasmin (Cp) in cement workers occupationally exposed to silica dust as biomarkers of exposure rather than biomarkers of effect for silicosis.

**METHODS:**

Plain chest X-ray & pulmonary functions were done for 30 silicotic and 42 non-silicotic workers and 42 controls. CT scan was done for the exposed groups. Serum levels of Cu, Cp and ACE were estimated.

**RESULTS:**

The results showed a higher significant difference between the exposed groups and controls, and between the two exposed groups regarding the mean levels of all measured biochemical parameters. The pulmonary functions were significantly lower among silicotic workers than controls and non-silicotic groups. There was a significant positive correlation between duration of employment and serum ACE and Cu.

**CONCLUSION:**

Since respirable dust exposure-linked lung fibrosis disease is non-curable, the biochemical parameters (Cu, ACE and Cp) can be used as exposure biomarkers to silica dust, providing a better way for early diagnosis of this deadly disease. Down regulating the inflammatory responses could potentially reduce the adverse clinical pulmonary effects of air pollution.

## Introduction

Silica dust is widely prevalent in the atmosphere and is more common than the other types of dust. Inhalation of various forms of free crystalline silica or silicon dioxide results in a spectrum of pulmonary diseases known as silicosis, thus making silicosis the most frequently occurring pneumoconiosis [1].

Mining, quarrying, tunneling, foundry work, glass manufacture, abrasive blasting, ceramic and pottery production, and cement production are industries and occupations having the potential for silica exposure [2].

Clinical diagnosis of silicosis depends on the detection of radiological abnormalities, which are a late (10 to 15 yr following exposure) and irreversible manifestations of the disease. Elucidation of the mechanisms of action of crystalline silica-induced fibrosis has contributed greatly to the identification of a number of biological responses that are involved in the pathogenesis of silicosis [3].

Histologically, silicosis is characterized by hyalinized and fibrotic nodules, thickening of alveolar interstitium, and accumulation of inflammatory cells such as alveolar macrophages (AM) and lymphocytes. The pathogenesis of silicosis has been related to the accumulation of inflammatory cells that produce fibrogenic and inflammatory cytokines and growth factors. AM are thought to be key inflammatory cells in silicosis, since they produce most of these fibrogenic factors in silicotic lung [4].

Gaensler and Carrington [5] stated that 9.6 - 18.1% of individuals having pathological evidence of interstitial lung disease may have a normal chest radiograph. Similarly the lung function tests also reveal the changes in the advanced stages [6]. Computed Tomography (CT) has recently been introduced for the diagnosis of pneumoconiosis. CT detects finer anatomical structure than radiography; it is expected to increase the sensitivity of diagnostic measures for this disease. But it could not get popularity because of its cost [7]. Hence, there is a need to develop a biomarker for early detection of silicosis.

The disease is irreversible and incurable, and only preventive steps such as job rotation, use of personal protective equipment, etc., remain solutions to the problem. Under such a situation, early diagnosis or prediction may become very useful to control the disease. Biomarkers are biological parameters in blood serum whose values are changed with deposition of dust in the lung and onset of lung fibrosis. They can help in the early diagnosis and prognosis of silicosis before it is actually diagnosed by the conventional X-ray technique and lung function tests. There are several reasons why such emphasis is currently placed on biomarkers. Biomarkers may enhance the diagnostic accuracy of occupational and environmental illness, and ultimately result in prevention of diseases. In addition, biomarkers are likely to enhance the understanding of the dose-response relationship between exposure to a hazard and an illness. Ultimately, their use may help to evaluate the effectiveness of various control measures [8].

In addition, it is also of importance to assess those biological responses before the threshold burden of silica in the lung has been exceeded. It has recently been shown that once this threshold has been exceeded, silica-induced pulmonary disease progresses without further exposure to silica [3].

ACE is a peptidyl dipeptide hydrolase in the renin–angiotensin system, converts angiotensin-I into the potent vasopressor angiotensin-II and inactivates the vasodilator bradykinin, which is the product of the kallikrein–kinin enzyme system. ACE is located mainly on the luminal surface of vascular endothelial cells [9] but is also present in monocyte–macrophage cells [10]. In silicosis and other dust-related lung fibrosis, both the endothelial cells and macrophages were considered to be the source of increased serum ACE levels [11].

The increase in the activity of ACE in bronchoalveolar lavage (BAL) fluid and serum was considered to be a marker of lung injury in a number of pulmonary diseases because its concentration in the serum is only a very small fraction of the total body ACE activity [12].

Cu has a fibrogenic property [13]. As the primary pathologic changes in silicosis include fibrosis and the proliferation of collagen tissue in the lungs there could be a possible association between silicosis and raised levels of serum Cu. Studies have reported elevated levels of serum Cu in silicotics [14, 15]. Although the mechanism of increase in serum Cu is still not understood. However, it has been suggested that an increase in Cp levels in silicosis, which contains eight Cu atoms may be responsible for such an increase [16]. Moreover, both parameters were observed to increase in the overt cases of silicosis with severe grades of profusion [17].

The aim of the current study was to measure serum levels of ACE, Cu and Cp in cement workers exposed to respirable dust containing free silica. Also, to address the importance for using these biomarkers as an early warning signal for risk of future adverse health outcomes, before it is actually diagnosed by the conventional X-ray technique and lung function tests used for diagnosis of dust-linked diseases.

## Materials and Methods

### Subjects

This work was designed as a cross-sectional controlled study. Three groups were included. The study was conducted at a cement factory located in Helwan district, Cairo Governorate, Egypt. Thirty silicotic workers from different departments of the cement factory (quarrying, crushing, milling and packing processes) were included in the study. Those cement workers were previously diagnosed as silicosis by the Occupational Diseases Committee, Health Insurance Agency, Cairo and they had occupational disability ratio (ranged from 5 to 25%) and their chest X-rays confirmed by High Resolution Computerized Tomography scan (CT) revealed that all the opacities were of p/p category and of 1/0, 1/1, 1/2 and 2/1 profusion grades.

Forty two non-silicotic workers were randomly chosen from the same departments of the cement factory. Subjects with duration of exposure with average 10 years were included in this study. Their chest X-rays and CT scan examinations revealed that all of them were free of silicosis. None of the exposed workers used any personal protective equipment as masks or respirators during their job. Forty two non-smokers male administrative workers with mean age 39.4 ± 5.3 years, who have never been involved in cement industry, were chosen as a control group (with free chest x-ray).

### Methods

1. Written informed consent was taken from each subject to confirm his voluntary participation in this study. The consent was signed by the principal investigator and the participant. All workers were interviewed to complete questionnaire sheet (personal data, smoking habit, detailed current and previous occupational history to find out work-related symptoms and past medical history of chronic illness). Subjects with chronic inflammatory diseases such as liver disease, rheumatoid arthritis, diabetes, hypertension, and thyroid disease were excluded from the present study.
2. Chest examination was done for all studied workers.
3. Plain chest X-ray: Full sized (14” × 14” inches) roentgenograms posterior-anterior view was done for all the studied groups.
4. High Resolution CT scan were performed for the exposed workers, and the roentgenographic findings were classified according to the International Labor Organization (I.L.O) classification of pneumoconiosis [18].
5. The pulmonary functions were measured using Spirovit SP-10 (Maker Schiller AG, Switzerland). After calibrating the spirometer according to the procedure given in the catalog, at least three acceptable valid and reproducible (within 5%) spirograms were taken. The readings showing the highest value were recorded. Spirometry prediction equations for Whites [19] automated in the spirometer were used. Recorded spirometric parameters included percent predicted forced vital capacity (% pred FVC), % pred forced expiratory volume in 1^st^ second (% pred FEV1) and FVC/FEV1 ratio.
6. Laboratory Investigations:
  Blood sample was collected from exposed workers and the controls, and centrifuged for the separation of serum, which was kept frozen until analyzed for serum copper, ceruloplasmin and ACE.

- Serum Cu was measured by coloremitric determination without deproteinization. Copper dissociated from proteins, gives with Di-Br-PAESA a stable colored complex, whose intensity of color is proportional at the concentration of copper in the sample [20]. The kit was purchased from BioSTC Egypt. Normal values (80-140 µg/dl).

- Serum Cp was assessed by radial immune-diffusion assays, employing Biocientifica S.A. - Argentina (ceruloplasmin) plate. The procedure is based on immune-precipitation in agarose between an antigen and its homologous antibody. Antigen diffuses radially out of the well into the surrounding gel-antibody mixture, and a visible ring of precipitation forms where the antigen and antibody reacted. A quantitative relationship exists between the ring diameter and antigen concentration [21]. Normal values (15-57 mg/dl).

- The serum ACE activity was measured by the spectrophotometric method; the synthetic tripeptide substrate used was N-[3-(2-furyl) acryloy]-L-phenylalanylglycylglycine (FAPGG). The method is based on the principle of hydrolysis of FAPGG to furylacryloylphenylalanine and glycylglycine. Hydrolysis of FAPGG results in a decrease in the absorbance at 340 nm. The ACE activity in the sample is determined by comparing the sample reaction rate to that obtained with the ACE calibrator (22). The kit was purchased from BEN-Biomedical Enterprise S.r.l. milano-Italy. Normal values (20-70 U/L).

Data entry and statistical procedures were performed using SPSS18 program.

## Results

All eligible workers with silicosis (n = 30) from different departments of the cement factory (quarrying, crushing, milling and packing processes) with mean duration of 26.8 ± 5.0years were included in the study. A non-silicosis group (n = 42) with mean duration of exposure 10.7 ± 3.3 years was randomly chosen from the same work departments. Although there was significant difference between the silicosis and non-silicosis group regarding the age (52.2 ± 4.6 and 37.7 ± 6.4 years respectively), the non-silicosis group was comparable to the silicosis group as regards the other potential confounders such as gender (males), smoking habit (ever smokers 24/42 (57.1%) and 18/30 (60.0%) respectively), socioeconomic and educational levels.

Table 1 show a higher significant difference between the exposed groups both silicosis and non silicosis compared with the controls and between the silicosis group and non silicosis as regards the mean levels of all measured serum biochemical parameters Cp, Cu and ACE. It was found that 40% (12/30) of the silicosis group showed higher level of Cu and 80% (24/30) showed higher level of ACE than normal values. While 4.8% (2/42) of the non-silicosis group showed higher level of ACE than normal values.

**Table 1:** The mean values of measured serum biochemical parameters of the studied groups

		N	Mean	Std. Deviation	F-ratio	ANOVA P-value	LSD
Serum ACE (U/L)	C	42	20.34	5.03	53.06	P< 0.0001	(N,S)
	N	42	50.33	12.45			(C,S)
	S	30	108.27	47.15			(C,N)
Serum Copper (μg/dl)	C	42	46.71	3.59	88.65	P< 0.0001	(N,S)
	N	42	81.01	5.13			(C,S)
	S	30	130.55	35.80			(C,N)
Serum Ceruloplasmin (mg/dl)	C	42	13.19	5.12	6.34	P< 0.005	(S)
	N	42	15.61	8.58			(S)
	S	30	21.95	10.16			(C,N)

C = control; N= non-silicosis; S = silicosis.

**Figure 1:**
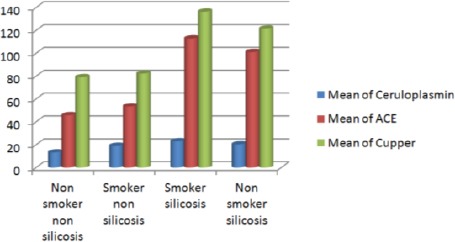
*Effect of smoking and silicosis on different biological parameters*.

Concerning the effect of smoking on the levels of serum Cp, Cu, and ACE in both studied exposed groups; there was no significant difference between the smokers and the non-smokers subgroups in both the silicotics and non-silicotics. Mean levels of serum Cp, Cu and ACE were found to be higher in silicotic workers compared to non-silicotic either non-smoker or smoker (Table 2, Fig. 1).

**Table 2:** Effect of smoking and silicosis on different biological parameters

	Non- smoker Non-silicosis (A) N=18		Smoker Non- silicosis (B) N=24		Non- smoker Silicosis (C) N=12		Smoker silicosis (D) N=18			ANOVA

	Mean	± SD	Mean	± SD	Mean	± SD	Mean	± SD		
Serum ACE (U/L) LSD	45.94 (C,D)	10.70	53.63 (C,D)	13.08	101.12 (A,B)	61.03	113.05 (A,B)	38.71	9.6	0.000

Serum Copper (μg/dl) LSD	79.17 (C,D)	4.77	82.39 (C,D)	5.15	121.61 (A,B)	30.38	136.51 (A,B)	39.58	13.5	0.00

Serum Ceruloplasmin (mg/dl) LSD	13.3 (D)	5.6	19.1	11.5	20.4	10.9	23.1 (A)	9.9	1.86	0.159

The percentage of impaired pulmonary function was significantly higher in the silicosis group compared to non silicosis 93.3% (28/30) and 61.9% (26/42) respectively (p < 0.05). It was found that odds ratio of silicosis group having impaired pulmonary function was 8.62 times (0.94-78.67) the non-silicosis.

Table 3 showed that the percent pred FVC (p < 0.001), the percent pred FEV_1_ (p < 0.001) and FEV_1_/FVC ratio (p < 0.05) were significantly lower among the silicosis workers than the control and the non-silicosis group. In the silicosis group, all workers had combined restrictive & obstructive ventilatory function impairment.

**Table 3:** Parameters of ventilatory functions among studied groups

		N	Mean	Std. Deviation	F-ratio	P-value	LSD
% pred FVC	C	42	88.24	16.32	16.894	0.000	(C,S), (N,S)

	N	42	83.23	16.23			

	S	30	57.00	14.26			

% pred FEV_1_	C	42	97.05	21.37	14.199	0.000	(C,S), (N,S)

	N	42	90.00	18.78			

	S	30	61.23	17.74			

FEV_1_/FVC	C	42	0.99	0.21	3.459	0.047	(C,S), (N,S)

	N	42	0.98	0.28			

	S	30	0.88	0.19			

C=control; N=non-silicosis; S=silicosis

Table 4 showed that there was a high significant difference between mean levels of serum Cp, Cu and ACE among workers with impaired lung functions and silicosis in comparison with workers with impaired lung functions and non silicosis. There was no correlation between measured biochemical parameters and the pulmonary functions tests (not tabulated).

**Table 4:** Mean and standard deviation of serum ceruloplasmin, ACE and copper in relation to silicosis condition among studied participants with impaired lung functions

	Groups with impaired lung functions		P value
	Silicosis (93.3%)	Non-silicosis (61.9%)	
	Mean ± SD	Mean ± SD	
Serum ACE (U/L)	110.87 ± 47.81	57.54 ± 10.09	0.001**
Serum Copper (μg/dl)	129.04 ± 36.66	83.47 ± 4.58	0.02*
Serum Ceruloplasmin (mg/dl)	24.5 ± 10.6	15.2 ± 8.7	0.000**

** p< 0.01

* p< 0.05.

There was no significant relationship between the degree of opacity in silicosis workers and smoking. As, smokers and non smokers with p opacity were (77.2% and 83.3% respectively), while smokers and non smokers with pq opacity were (22.8% and 16.7% respectively). They are both independent variables. The studied serum biological parameters ACE and Cu were higher in workers with pq & p opacities than the non-silicosis workers. Also, they are higher in workers with pq opacities than those with p opacities (Table 5).

**Table 5:** Relation between the categories of opacities and the studied biological parameters

	Workers with opacities (N=30)				Non-silicosis Workers N=42	ANOVA	P value

	Workers with p opacities N=24		Workers with pq opacities N=6				

	Mean	± SD	Mean	± SD			
Serum ACE (U/L)	98.77	37.64	146.28	71.13	50.3 ± 12.5	37.376	0.000**

Serum Copper (μg/dl)	106.96	11.06	136.44	37.68	81 ± 5.1	68.915	0.000**

Serum Ceruloplasmin (mg/dl)	15.6	8.6	16.3	9.3	12.3 ± 4.8	1.608	0.208 NS

** p< 0.01; NS = non significant.

There was a significant positive correlation between duration of employment and ACE and Cu (Table 6).

**Table 6:** Correlation between the duration of employment and the measured biochemical parameters in cement workers

Number of exposed workers (72)		Duration of employment
Serum ACE	r	0.518**

	p value	0.001

Serum Copper	r	0.709**

	p value	0.000

Serum Ceruloplasmin	r	0.132

	p value	0.443

** p< 0.001.

## Discussion

Serum ACE and copper levels are elevated in fibrotic lung diseases. They can be used as potential biomarkers of exposure in silicosis not only in the classical cases of silicosis but also in the covert cases of silicosis so that the preventive measures can be taken [8].

Serum ACE is considered a useful biomarker to monitor lung damage from exposure to crystalline silica. The rise in serum ACE activity due to hypertension can be ruled out as the blood pressure measurement of workers revealed normo-tension. Human studies have produced contradictory results. Stone workers in the Gwalior region exposed to silica dust had increased serum ACE level [23]. Increased serum ACE activities were reported in silicosis patients compared to control subjects, but this activity did not reflect the severity of the disease as determined by chest x-ray changes and respiratory function tests, did not give further information on the progression of the disease [24, 25]. Investigation of Coal Workers Pneumoconiosis (CWP) showed elevated serum ACE activity in CWP compared to control subjects but no correlation was seen with the progression of the disease [26]. However, other studies confirmed an elevation in serum ACE activities in silicosis patients. They found an association between the serum ACE activity and the roentgenographic severity of fibrosis [27, 28].

In the current study, the significant increase in the ACE level among these exposed workers compared to the controls and the higher mean levels of serum ACE in non-smoker and smoker silicotic workers compared to non-smoker and smoker non-silicotics, confirmed that the exposure to silica dust had a major role in this increase of ACE. This increase reflects the severity of the disease as determined by chest x-ray changes (Table 5) and respiratory function tests (Table 4). Considering the pulmonary function status, serum ACE level, was higher in those with abnormal pulmonary function and silicosis than those with abnormal pulmonary function and free from silicosis. Those findings were in concordance with Bucca et al., [25] and Tiwari et al., [29].

Romano et al., [30] explained that elevated serum ACE levels can be attributed to the fibrotic involvement of lung tissue; including capillaries as the endothelial cells in the capillary bed have high angiotensin-1-converting enzyme content. Human AM are also known to contain ACE, and the mononuclear phagocyte cell line is considered the primary source of serum ACE in sarcoidosis [31]. The cytotoxic effect of silica particles on macrophages leading to their rupture and loss of the cytoplasmic contents, seem to be crucial in the development of silicosis [32]. It is likely that the serum ACE level in silicosis, at least partly, reflects the accumulation and the increased degradation of macrophages; this indicates that macrophages may be a source of serum ACE in silicosis.

There is an increasing evidence that AM plays a key role in local lung and systemic inflammatory responses induced by exposure to ambient particulate matter (PM). In the lung, AM contributes to the magnitude and the nature of the inflammatory response by interacting with other lung cells such as bronchial epithelial cells and dendritic cells in an effort to process and clear the PM from the lung. These macrophages also produce the mediators that are associated with the systemic inflammatory response induced by PM exposure, and recent studies support the concept that these systemic mediators translocate from the lung tissues into the circulation [33]. The increased IL-1β production is mediated by the nucleotide-binding domain and leucine-rich repeat protein 3 (NLRP3) inflammasome that spreads the local inflammatory response by interacting with resident dendritic cells residing within or near the epithelium, initiating, and maintaining an adaptive immune response [34].

The elevated level of serum Cu in silicosis was similar to the findings of earlier studies [16-35]. The elevated levels of serum Cu due to smoking as reported in few studies [36, 37] can also be ruled out, as in the current study, smoking had no effect on levels of serum Cu and Cp in both exposed studied groups, as there was no significant difference between the smokers and the non-smokers subgroups in non-silicosis and the silicosis. While the mean levels of serum Cu and Cp were found to be higher in silicosis workers compared to non-silicosis either non-smoker or smoker which confirmed that the exposure to silica dust had a major role in this increase.

Approximately 90–95% of the total amount of Cu in blood serum is strongly protein-bound, mostly with α2–globulin (Cp). The concurrent increase of serum Cp level during induced inflammatory conditions suggest the involvement of serum Cp as one of the body’s inbuilt defensive mechanism against noxious responses or inflammation [38]. It is suggested that the increased serum Cp levels may be a complimentary factor associated with inflammatory conditions [39]. Serum concentration of Cp increases in the presence of inflammation or infection, as a consequence of its enhanced synthesis. This is mostly due to the Cp production in hepatocytes, stimulated by proinflammatory interleukins, such as IL-1 and IL-6. During the inflammatory process, the production of cytokines is increased. Both interleukins: IL-1 and IL-6 are responsible for hepatocytes stimulation to increase the synthesis and secretion of Cp to the blood serum. Cp transfers Cu from hepatocytes to the blood serum (and further, to the tissues) [38]. This may possibly explain the elevated concentration of this bioelement (Cu), as well as the increased Cp level, in serum of our study workers. When pulmonary function tests were categorized according to respiratory diseases, serum Cu levels were found to be higher among those having silicosis compared with those free from diseases. Our findings agreed with those of Tiwari et al., study [37].

In our study, serum Cu and Cp levels were higher in workers with lung opacities than the non-silicosis workers free of lung opacities, and in workers with pq lung opacities than those with p lung opacities. Inflammatory pathway plays pivotal role in development and progression of the disease. These findings were confirmed by Strecker et al., [40] results who found that levels of serum Cu and Cp in patients with rheumatoid arthritis correlated with the presence of the inflammatory process. The Cu and Cp concentrations increased proportionally to clinical activity. This is considered to be a form of organism’s protective response, since the Cp acts as an antioxidant [40].

We found a significant positive correlation between duration of employment and ACE and serum Cu. This can be attributed to the increased cumulative exposure of workers to the silica dust. This finding disagree with that of Tiwari et al. [29] who observed no association between serum ACE activity and duration of exposure. Pandey and Agarwal [41] suggested that with increasing duration of exposure, the chance for developing silicosis increases, and thus fibrosis of lungs due to silicosis is accompanied by increases in Cp as well as Cu levels in blood serum. In contrast, a study which was conducted by Tiwari et al. [37] on 134 workers of quartz stone crushing units to assess serum Cu activity did not found a significant correlation between mean serum Cu levels and the duration of exposure.

In conclusion, since respirable dust exposure-linked lung fibrosis disease is non-curable, there is a need to develop a biomarker of exposure rather than developing a biomarker of effect for silicosis because of irreversible nature of the disease. So in the present study the biochemical parameters (Cu, ACE and Cp) can be used as exposure biomarkers to silica dust, providing a better way for early diagnosis of this disease.

Improvement in industrial hygiene, techniques such as wet drilling, complete enclosed system, mechanization; efficient ventilation systems (artificial, natural, local and exhaust ventilation) and usage of personal protective devices can prevent silicosis to some extent. The problem of respirable dust exposure-linked lung fibrosis should be dealt with through a medico-socio-engineering approach, to provide an optimum solution to this disease. It is reasonable to suppose that treatment of the local and systemic inflammatory responses of AMs, induced by PM exposure, would be of benefit in reducing the adverse clinical pulmonary effects of air pollution. Further future studies are recommended to overcome the current study’s limitation of relatively small sample size.
